# Tobacco Harm Reduction with Vaporised Nicotine (THRiVe): The Study Protocol of an Uncontrolled Feasibility Study of Novel Nicotine Replacement Products among People Living with HIV Who Smoke

**DOI:** 10.3390/ijerph14070799

**Published:** 2017-07-18

**Authors:** Stephanie Bell, Judith Dean, Charles Gilks, Mark A. Boyd, Lisa Fitzgerald, Allyson Mutch, Peter Baker, Graham Neilsen, Coral E. Gartner

**Affiliations:** 1 School of Public Health, University of Queensland, Cnr Wyndham Street and Herston Road, Herston, QSD 4006, Australia; j.dean4@uq.edu.au (J.D.); c.gilks@uq.edu.au (C.G.); l.fitzgerald@sph.uq.edu.au (L.F.); a.mutch@sph.uq.edu.au (A.M.); p.baker1@uq.edu.a (P.B.); Graham.Neilsen@health.qld.gov.au (G.N.); c.gartner@uq.edu.au (C.E.G.); 2 Lyell McEwin Hospital, University of Adelaide, Haydown Road, Elizabeth Vale, SA 5112, Australia; mark.boyd@adelaide.edu.au; 3 Sexual Health and HIV Service, Metro North Hospital and Health Service, Biala, 270 Roma Street, Brisbane, QSD 4000, Australia

**Keywords:** smoking, tobacco, HIV, harm reduction, vaporised nicotine, VNPs, e-cigarettes, feasibility

## Abstract

Smoking is a leading cause of morbidity and premature mortality among people living with HIV (PLHIV), who have high rates of tobacco smoking. Vaporised nicotine products (VNPs) are growing in popularity as a quit aid and harm reduction tool. However, little is known about their acceptability and use among PLHIV. Using a pragmatic, uncontrolled, mixed methods design this exploratory clinical trial aims to examine the feasibility of conducting a powered randomised clinical trial of VNPs as a smoking cessation and harm reduction intervention among vulnerable populations, such as PLHIV who smoke tobacco. Convenience sampling and snowball methods will be used to recruit participants (N = 30) who will receive two VNPs and up to 12 weeks’ supply of nicotine e-liquid to use in a quit attempt. Surveys will be completed at weeks 0 (baseline), 4, 8, 12 (end of treatment) and 24 (end of the study) and qualitative interviews at weeks 0 and 12. As far as we are aware, this feasibility study is the first to trial VNPs among PLHIV for smoking cessation. If feasible and effective, this intervention could offer a new approach to reducing the high burden of tobacco-related disease among PLHIV and other vulnerable populations.

## 1. Introduction

Antiretroviral therapy has reduced HIV-related morbidity and increased the life expectancy of people living with HIV (PLHIV) [1]. However, the potential health gains from advances in HIV treatment have been lessened by increased rates of non-AIDS morbidity and mortality in PLHIV [2]. While some evidence exists that this may, in part, be related to increased rates of inflammation in PLHIV despite full suppression of viral replication in those successfully receiving antiretroviral therapy [3], this increased burden of disease may relate to higher rates of adverse health behaviours in PLHIV, for instance tobacco smoking [4,5,6,7]. With tobacco smoking rates two to three times greater than the general population, smoking is now a significant risk factor for premature mortality among PLHIV [7,8,9]. More years of life are now lost from smoking than from HIV itself [9] in this population.

Many PLHIV want to quit smoking [10] and most PLHIV who smoke have made previous unsuccessful quit attempts [11,12,13]. However, evidence suggests that many currently available cessation products such as nicotine replacement therapy (NRT) and smoking medications have low acceptability among this population [14], which is reflected in the low levels of reported past use of these smoking cessation interventions [15]. The vast majority of PLHIV who attempt to quit smoking experience substantial difficulty converting a quit attempt into long-term abstinence [16]. Many smoking cessation interventions trialled in PLHIV have been effective at achieving short-term abstinence, but few have achieved long-term effects, with relapse to smoking common after the intervention is stopped [16]. Interventions that have been evaluated include both behavioural (e.g., counselling, mobile phone text messaging) and pharmacological support (e.g., nicotine patches) with an abstinence-focussed goal [16]. Given the limited success in attaining long-term abstinence, new approaches are needed.

Tobacco harm reduction (THR) may offer a complementary strategy to the cessation model to reduce tobacco-related harms [17] among PLHIV. THR aims to reduce tobacco-related morbidity and mortality without necessarily requiring complete abstinence [18]. Harm reduction approaches have been well accepted and highly successful in the HIV field for prevention of HIV transmission via sexual contact or injecting drug use [19,20,21]. Harm reduction has not however been applied to reducing tobacco-related harms among PLHIV.

Although nicotine is the addictive agent in tobacco cigarettes, it is not responsible for the majority of tobacco-related harm [22]. Rather, it is the carcinogenic tars, toxic gases and fine particulate matter present in tobacco smoke that are the main contributors to the development of tobacco-related cancers, respiratory diseases and cardiovascular disease. One potentially promising THR strategy is encouraging smokers to switch from tobacco smoking to using a non-smoked nicotine product, such as NRT, smokeless tobacco, or vaporised nicotine products (VNPs), also known as e-cigarettes or electronic nicotine delivery systems. VNPs are electronic devices that convert nicotine e-liquid into an aerosol by heating it. The aerosol, commonly termed “vapour” [23] is inhaled by the user by puffing on the mouthpiece, which is known as “vaping”. While long-term nicotine use may still convey some health risks, these are likely to be a small fraction of the risks associated with continued smoking. For example, the risks of vaping are reported as being unlikely to exceed 5% of the risks of smoking [24,25].

NRT is widely available and its long-term use for THR is endorsed in some clinical guidelines [17], however relatively few smokers use existing NRT products as a long-term substitute for cigarettes [26], suggesting low consumer-acceptability. VNPs may be more acceptable and suitable to smokers for long-term substitution as their mode of use replicates some aspects of smoking. For example, individuals’ vaping can self-titrate their nicotine consumption [27] with their nicotine delivery profile closer to smoking [28].

While the evidence base for VNPs as a smoking cessation aid is still developing, clinical trials and observational data suggest that VNPs increase tobacco cessation success compared to no cessation aid, are at least as effective as other nicotine replacement products (e.g., patches) [23,29], and may also help people reduce tobacco smoking [25,29]. In the UK, where VNPs are available as consumer products [30], they are widely used as unapproved cessation aids [25] and sales surpass that of NRT [31], and are the most commonly used smoking cessation aid [25]. This suggests that the general population of smokers prefer VNPs to currently approved cessation aids. This feasibility study will explore whether VNPs are acceptable to PLHIV who smoke.

## 2. Materials and Methods

### 2.1. Trial Aim and Objectives

The overall aim of this exploratory clinical study is to examine the feasibility of a randomised trial of VNPs as a smoking cessation or THR intervention in PLHIV who smoke. The primary objective of this study is to evaluate the acceptability and use of two vaping devices (Innokin Endura T18^®^ and Innokin Endura T22^®^) with and an e-liquid (Nicophar: 12 mg/mL of nicotine in glycerol and purified water) for quitting smoking or reducing the number of cigarettes smoked per day, among PLHIV, who smoke 5 or more cigarettes per day.

The secondary objectives will complement the primary study objective by assessing changes in smoking behaviour, including:

1. Measuring quit attempts.
2. Measuring cigarettes smoked per day.
3. Evaluating short-term (7-day) abstinence from cigarette smoking (at 4, 8, 12 and 24 weeks post treatment initiation).
4. Evaluating medium-term abstinence from cigarette smoking (continuous, abstinence from tobacco smoking for at least 8 weeks), measured at 12 and 24 weeks post treatment initiation.
5. Obtaining feedback on the intervention (including, printed information, investigational devices and medicine).
6. Obtaining data to accurately describe the sample (e.g., demographic features, history of smoking and quitting), knowledge of health impacts, their level of quitting motivation, quit self-efficacy and level of nicotine dependence.
7. Monitoring adverse events and contribute to the literature on the safety of vaping as a method to quit smoking.

### 2.2. Design

**Figure 1:**
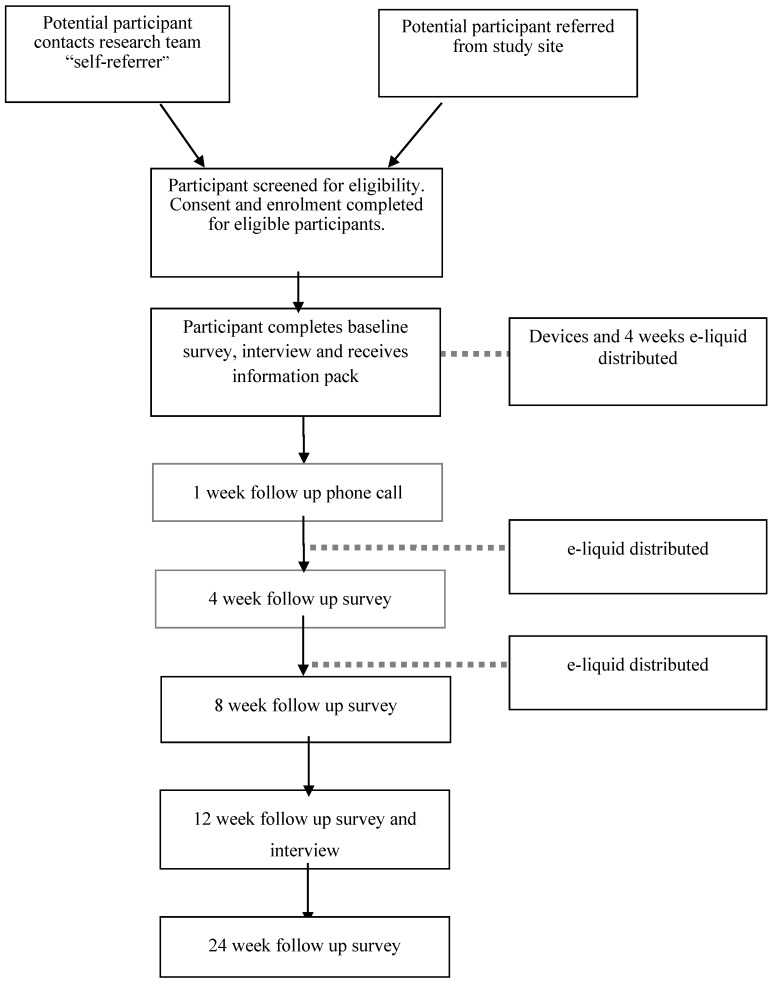
Study flow diagram.

The study will use a mixed methods design. Both quantitative survey data with qualitative data obtained in semi-structured interviews will be collected throughout the study. A mixed methods approach will be used to obtain a richer understanding of participants’ experiences of using the intervention and in recognition of the complexity of behaviour change, incorporate the exploration of the social context in which the participants will be using the intervention [32]. Acceptability of the trial intervention, rather than efficacy is the primary objective of the study and therefore no control arm will be used (see Figure 1).

### 2.3. Research Ethics

All subjects will provide informed consent before participating in the study and the study will be conducted in accordance with the Declaration of Helsinki. The study protocol is approved by the Metro South Hospital and Health Service, Human Research Ethics Committee (HREC/16/QPAH/693) and is ratified by the University of Queensland’s Human Research Ethics Committee (2016001749/HREC/16/QPAH/693).

### 2.4. Sample

#### 2.4.1. Participants

The study aims to recruit 30 participants from Brisbane, Australia, who meet eligibility criteria through convenience sampling and snowballing methods.

#### 2.4.2. Eligibility Criteria

- Inclusion criteria: diagnosis of HIV; aged 18 years, or over; smoke ≥5 cigarettes per day at the time of enrolment into the trial; have been smoking for at least 12 months; have capacity to consent and able to understand study instructions and procedures (e.g., sufficient English language ability); willing to attempt to quit tobacco smoking after study enrolment.
- Exclusion criteria: participating in a smoking-cessation program; pregnant (measured by self-report, with confirmation by self-administered pregnancy test where there is doubt) or planning to become pregnant during trial participation period; breast-feeding (measured by self-report) or planning to be during trial participation period; experienced chest pain, or another cardiovascular event or procedure (e.g., heart attack, stroke, insertion of stent, bypass surgery) in the last month; being treated with oxygen therapy.

All inclusion and exclusion criteria will be measured by self-report, with the exception of capacity to consent which will be determined by the member of the research team conducting the consent and enrolment process.

#### 2.4.3. Recruitment

The study will be advertised through targeted settings such as community support organisations for PLHIV, and high HIV caseload general practitioner and public sexual health and HIV clinics. Potential participants will be able to contact researchers to complete an eligibility screen over the phone or self-administered online. Health practitioners of participating sexual health services may also screen and directly refer eligible participants for enrolment. Participants will be informed of the risks and benefits of participating in the study and provided an opportunity to ask questions. After consenting to participate in the trial, participants will be assigned a unique study identification number (in a re-identifiable format) and complete the baseline assessments.

### 2.5. Intervention Description

Each participant will receive an intervention pack consisting of the following.

1. Printed information
  - Information and instruction booklet. The booklet outlines instructions on how to use, store and handle the investigational products, information of vaping instead of quitting and information and links on quitting smoking resources supports (such as Quitline) and tips from vapers.
  - A “Positively Quitting” booklet. A booklet developed by Queensland Positive People to help PLHIV to quit smoking containing information such as the benefits of quitting and suggestions for managing cigarette cravings.
  - Copies of device user manuals.
  - A study wallet card confirming participation in the trial.
2. Investigational devices
  - One Innokin Endura T18^®^ vaporiser kit
  - One Innokin Endura T22^®^ vaporiser kit
  - Four spare coils
  - One wall charger
3. Investigational medicine
  - Ten 10 mL bottles of Nicophar^®^ 12 mg nicotine e-liquid

The T22 has both a larger tank (4 mL) and longer battery life (2000 mAh) than the T18. As a result, the T22 does not require refilling and recharging as regularly as the T18, however it is a larger and heavier device to accommodate these features. Receiving one of each device allows for one device to be used while the other is recharging. It also allows participants to pick the most practical device for their smoking pattern and situation. For example, the larger device may be more practical for heavier smokers or the smaller device may be used more away from the home as it is more discrete and portable. The devices were selected due to their ease of use, manufacturing quality and safety features (electrical safety cut-offs, leak-proof design).

The nicotine e-liquid used is manufactured to pharmaceutical quality and contains only glycerol, purified water and nicotine. While most regular vapers use flavoured e-liquids [33], an unflavoured e-liquid was selected because flavour preferences could vary substantially between participants. The selected nicotine concentration (12 mg/mL) is recommended by the manufacturer as the most appropriate strength to be used with the Innokin Endura devices and a common strength in use [34]. It is also similar to the amount of nicotine contained in other currently available products such as the Nicorette QuickMist Mouthspray (contains 13.6 mg/mL nicotine), a liquid nicotine product currently listed on the Australian Register of Therapeutic Goods for treatment of withdrawal from smoking tobacco. At baseline, participants will receive 10 × 10 mL bottles of nicotine e-liquid intended to be sufficient for the first four weeks based on an estimated maximum e-liquid use of 3.5 mL/day which is based on guidelines for medical practitioners prescribing liquid nicotine [34] and advice from the e-liquid supplier.

Participants set a “start vaping date” within one week of receiving their intervention pack and are contacted to address any problems or queries concerning the intervention products. The 12 weeks immediately following their “start vaping date” will be the “treatment period” of the trial in which participants will be asked to make a quit attempt by replacing as many tobacco cigarettes as possible (if not all) with VNP use. Participants will be contacted at approximately weeks three and seven to determine how much e-liquid they require for weeks 5–8 and 9–12 respectively. New coils will also be provided as needed with each e-liquid supply. Participants will self-administer the e-liquid with the vaping devices, ad libitum over the 12 week treatment period to relieve nicotine cravings.

### 2.6. Data Collection Points and Outcome Measures

A battery of measures to assess primary and secondary objectives will be conducted at baseline and weeks 4, 8, 12 and 24. Quantitative data will be collected at all-time points and semi-structured interviews at baseline and 12 weeks (end of treatment) (see Table 1).

#### 2.6.1. Primary Outcome Measures

The primary objective is to evaluate acceptability and use of trial products, which will be measured using quantitative survey tools designed for these purposes and qualitative analysis of interviews with participants. Existing measures will be used, in addition to survey items and interview guides developed for the study to explore:

1. Familiarity and experience with VNPs
2. Attitudes toward VNPs
  - Acceptability of trial products and associated reasons (e.g., impact on cravings, assisting with quitting, ease of use)
  - Comparison of perceptions and use of trial products with traditional smoking cessation pharmacotherapy (e.g., varenicline, bupropion and NRT).
3. Level of interest in short and long-term use of VNPs
4. Nature of product use
  - Quantity of e-liquid and coils used
  - Locations, when used and how often, reasons for use, ease of use
  - Barriers and facilitators to VNP use, such as adequacy for reducing withdrawal

#### 2.6.2. Secondary Outcome Measures

The secondary outcome measures will include:

1. Number of quit attempts
2. Tobacco cigarettes smoked per day (CPD)
3. Abstinence measures:
  - Short-term abstinence or 7 days point prevalence (defined as having not smoked any tobacco in the previous 7 days at assessment) at weeks 4, 8, 12 and 24.
  - Medium-term abstinence from tobacco smoking measured by continuous abstinence from tobacco smoking for at least 8 weeks post quit date, with no more than five (tobacco) cigarettes from the start of the abstinence period (measured at weeks 12 and 24).
4. Descriptive measures of the sample:
  - Demographics
  - History of tobacco smoking and quitting
  - Knowledge of health effects of smoking and nicotine
  - Motivation to quit
  - Quitting self-efficacy
  - Fagerstrom Test for Nicotine Dependence (FTND) [35]
  - A measure of behavioural dependence, using the Glover-Nilsson Smoking Behavioural Questionnaire (GN-SBQ) [36]
5. Measures of adverse events.

#### 2.6.3. Participant Reimbursement

Participants will be reimbursed for the inconvenience and time involved in completing data collection throughout the study. Participants will be reimbursed with cash, as follows, with greater amounts at baseline and week 12 reflecting the longer duration of the data collection at these time points:

- Baseline survey and interview: $50
- Week 4 survey: $20
- Week 8 survey: $20
- Week 12 (end of treatment) survey and interview: $50
- Week 24 (final follow-up) survey: $20

Payment will be made in Australian dollars and via bank deposit into the participants’ nominated bank account.

### 2.7. Safety and Adverse Events

Accidental overdose with the trial products is highly unlikely. Trials involving concomitant use of nicotine products and smoking have found that this use is safe and does not lead to nicotine intoxication [36,37,38]. Dual use of VNP and tobacco smoking is also common [37] particularly when transitioning from smoking to vaping. Evidence suggests that people who vape self-titrate [27] and accidental overuse is unlikely due to unpleasant (but not serious) effects (e.g., nausea). The resupply of e-liquid will also be adjusted to ensure participants have no more than 10 bottles of e-liquid at any one point in time to avoid stockpiling of unused e-liquid. Participants will be advised on how to store and handle the product safely (e.g., keep out of the reach of children, avoid contact with eyes and skin).

Each participant will be monitored for adverse events that occur between consent and the final follow-up (at 24 weeks post intervention start date). Participants will also be encouraged to spontaneously report any health issues or unusual symptoms and seek medical treatment for any immediate medical concerns. All adverse events will be recorded.

### 2.8. Participant Attrition

We have incorporated a number of approaches in the study design to minimise attrition. These include establishing good relationships with participants at enrolment and maintaining regular contact, using multiple methods to contact participants (email, phone and posted letter), offering a choice of setting for face-to-face interviews (university or PLHIV community-based organisation) and providing reimbursement for participants’ time spent completing follow-ups and to cover transport costs associated with attending face-to-face interviews. Retention rates at each follow-up point will be reported with the study findings.

### 2.9. Data Management and Analysis

Data necessary to complete a CONSORT (Consolidated Standards of Reporting Trials) flow diagram will be recorded to adhere to CONSORT guidelines [39]. All data collected during the study will be entered (and stored) electronically on REDCap, a secure web based data storage and management application. Quantitative data will then be analysed in SPSS [40]. Descriptive statistics (counts, percentages and means) will be calculated for all quantitative results. A repeated measures design will be used to compare baseline and 12 weeks responses for: attitudes to VNP, interest in long and short-term use of VNP, measures of dependence (FTND and GNSBQ), knowledge of harms, quitting self-efficacy, cigarettes smoked per day and abstinence measures. No formal statistical tests will be conducted due to the small sample size.

Interviews will be audio recorded and transcribed (in de-identified format). At baseline interview, a participant’s feelings and beliefs about: smoking, quitting and quit smoking products (e.g., NRT) and services, VNPs and tobacco harm reduction will be explored. The second interview (post intervention, at 12 weeks) will discuss the same topics discussed during the baseline interview as well as their experience of using the VNP (such as what participants liked and did not like about the intervention, reactions of others and concerns) and how it compared to other quit attempts and quit smoking products they have used in the past. The two interviews together will allow greater understanding of participants’ experiences and how use of the intervention influenced opinions on smoking, quitting and tobacco harm reduction. Thematic analysis of qualitative data will be conducted and emerging themes identified, coded and categorised [41]. We will explore acceptability of the vaping devices and the e-liquid to determine if these will be suitable for use in the planned larger, randomised clinical trial.

## 3. Discussion

Many PLHIV want to quit smoking [10]. Continued high rates of tobacco smoking [6] and high rates of unsuccessful quitting [11,12,13] in conjunction with the deleterious health consequences of smoking for this vulnerable population [8,9] highlight the need for effective smoking interventions for PLHIV [16]. This study aims to test the feasibility of conducting a large randomised clinical trial of a tobacco harm reduction intervention with vaporised nicotine among PLHIV who smoke.

### 3.1. Strengths/Usefulness of Findings

This study will identify whether participants find the tested intervention acceptable and suitable for use. For an intervention to be successful, it must be viewed favourably by its target audience. Many PLHIV report not liking or using traditional NRT [14,15]. This feasibility study will provide important information to assist the planning of a large, randomised clinical trial to establish the efficacy of such an intervention. In particular, the trial will obtain valuable participant feedback on the investigational products and other intervention material, help researchers to understand the smoking behaviour and demographics of the population to be targeted and provide preliminary data on the impact of the intervention on smoking behaviour.

Clinical trials with vulnerable population groups can often suffer from high rates of attrition which can be a potential threat to the validity of the study results. In a previous smoking cessation intervention trial among PLHIV, only 52% of participants provided data at the 2-month follow-up although 62% completed the 4-month follow-up and 72% completed the 6-month follow-up [42]. We have addressed this potential limitation by incorporating a number of approaches in the design to minimise attrition. Data collected on retention at each follow-up from this feasibility study will assist with planning a larger RCT and provide information on which contact methods are most effective.

### 3.2. Innovation/Novelty and Strengths

This study will be the first (to our knowledge) to test VNPs among PLHIV in an extended trial (12 weeks). The mixed methods study design recognises the complexity of smoking-related behaviour change and is likely to be best suited to evaluating the acceptability of the intervention. The pragmatic approach to the intervention delivery (ad libitum use of the VNP) allows participants to use it in a way that is most suited to their individual needs and smoking behaviour. It also avoids the need to enforce strict intervention adherence criteria and overly artificial circumstances [43] which may help to reduce the high attrition rates seen in many other smoking intervention studies [16]. The use of mixed methodology will also provide a rich data set, highly valuable to not only interpreting the quantitative data but in refining and developing the planned larger trial so that the intervention meets the needs and preferences of vulnerable populations, such as PLHIV who smoke.

### 3.3. Limitations 

Measuring cigarettes smoked per day and short and medium-term abstinence are secondary objectives of this study. These objectives, are measured by self-report without a biochemical confirmation measure. Although the study would benefit from a biochemical confirmation measure, this is not possible due to the limited funding available, however this should be included in a future, larger efficacy trial of the tested intervention.

It may also be considered a limitation of the study that an unflavoured e-liquid is used. We are unable to offer participants a choice of flavour because only one type of liquid can be purchased because the limited funding available precludes the purchase of a range of flavours. There are a large number of e-liquid flavours available in countries that allow VNPs to be sold outside a therapeutic context [44]. There is substantial heterogeneity in flavour preferences among vapers and switching between flavours is also common practice [45]. Given the uncertainty about which flavour would be acceptable to the majority of participants and some concerns in the literature about the safety of inhaling certain flavours [46,47], an unflavoured/neutral product has been selected for this study. The acceptability of the unflavoured product will be explored in the qualitative interviews.

## 4. Conclusions

The results of this study will assist the planning of a larger, clinical trial to determine the effectiveness of a THR approach using VNPs to achieve long-term cessation and relapse prevention among PLHIV and other vulnerable populations experiencing premature mortality associated with tobacco smoking. This research is significant as it will contribute to the understanding of the potential for VNPs to be used to reduce tobacco-related harms globally and could lead to the development of a novel public health response to the high rates of smoking among PLHIV. Finally, it will contribute to policy discussion on how VNPs should be regulated to maximise their potential public health benefits while minimising risks.

## Figures and Tables

**Table 1:** Schedule of events.

Contact	Baseline					
Week (month)	0	1 (1)	4 (1)	8 (2)	12 (3)	24 (6)
Treatment Period (12 weeks)		X	X	X	X	
Device dispensation	X					
e-liquid dispensation	X		X	X		
1 week check in		X				
Measures: Primary Objectives						
Familiarity with vaporized nicotine products (VNPs)—survey	X					
Attitudes and use of VNPs—survey	X		X	X	X	X
Attitudes towards smoking, quitting and VNPs—interview	X				X	
Measures: Secondary Objectives						
Cigarettes per day (CPD)	X		X	X	X	X
Quit attempts	X		X	X	X	X
Abstinence measures			X	X	X	X
Demographics	X					
Smoking and quitting	X					
Knowledge of harms	X				X	
Quitting self-efficacy	X				X	
Fagerstrom Test for Nicotine Dependence (FTND)	X				X	
Glover Nilsson Smoking Behavioural Questionnaire (GNSBQ)	X				X	
Adverse events		X	X	X	X	X
